# Kriged and modeled ambient air levels of benzene in an urban environment: an exposure assessment study

**DOI:** 10.1186/1476-069X-10-21

**Published:** 2011-03-21

**Authors:** Kristina W Whitworth, Elaine Symanski, Dejian Lai, Ann L Coker

**Affiliations:** 1 Division of Epidemiology and Disease Control, University of Texas School of Public Health, Houston, TX, 77030 USA; 2 Division of Biostatistics, University of Texas School of Public Health, Houston, TX, 77030 USA; 3 Departments of Obstetrics & Gynecology and Epidemiology, University of Kentucky, Lexington, KY, 40536 USA

## Abstract

**Background:**

There is increasing concern regarding the potential adverse health effects of air pollution, particularly hazardous air pollutants (HAPs). However, quantifying exposure to these pollutants is problematic.

**Objective:**

Our goal was to explore the utility of kriging, a spatial interpolation method, for exposure assessment in epidemiologic studies of HAPs. We used benzene as an example and compared census tract-level kriged predictions to estimates obtained from the 1999 U.S. EPA National Air Toxics Assessment (NATA), Assessment System for Population Exposure Nationwide (ASPEN) model.

**Methods:**

Kriged predictions were generated for 649 census tracts in Harris County, Texas using estimates of annual benzene air concentrations from 17 monitoring sites operating in Harris and surrounding counties from 1998 to 2000. Year 1999 ASPEN modeled estimates were also obtained for each census tract. Spearman rank correlation analyses were performed on the modeled and kriged benzene levels. Weighted kappa statistics were computed to assess agreement between discretized kriged and modeled estimates of ambient air levels of benzene.

**Results:**

There was modest correlation between the predicted and modeled values across census tracts. Overall, 56.2%, 40.7%, 31.5% and 28.2% of census tracts were classified as having 'low', 'medium-low', 'medium-high' and 'high' ambient air levels of benzene, respectively, comparing predicted and modeled benzene levels. The weighted kappa statistic was 0.26 (95% confidence interval (CI) = 0.20, 0.31), indicating poor agreement between the two methods.

**Conclusions:**

There was a lack of concordance between predicted and modeled ambient air levels of benzene. Applying methods of spatial interpolation for assessing exposure to ambient air pollutants in health effect studies is hindered by the placement and number of existing stationary monitors collecting HAP data. Routine monitoring needs to be expanded if we are to use these data to better assess environmental health risks in the future.

## Background

Historically, there has been concern regarding the potential adverse human health effects of ozone, sulfur dioxide, nitrogen oxides, carbon monoxide, particulates, and lead. In 1971, the Clean Air Act was established under which National Ambient Air Quality Standards (NAAQS) were created to regulate ambient air concentrations of these six criteria pollutants [1]. A decline in ambient air concentrations of criteria pollutants has been observed since the induction of this Act [2], and more recently, the focus has shifted to hazardous air pollutants (HAPs), a class of 189 compounds, which are known or suspected to have adverse effects on health [3]. One HAP, benzene, is of particular concern due to its ubiquitous nature and ability to cause cancer in humans [4]. Although the general population is exposed to background levels of benzene, one of the major outdoor sources of personal exposure is vehicular exhaust; additionally, people living near chemical manufacturing facilities or oil refineries may also be exposed to elevated levels of benzene [4].

The potential for human exposure to benzene is well established, but quantifying exposure for population-based epidemiologic studies is problematic and requires immense resources. For this reason, researchers studying the health effects of ambient air levels of benzene often rely on proxy measures of exposure. One potential source of data is routine monitoring data. Tools like geographic information systems (GIS) and spatial interpolation methods such as kriging have helped to utilize these data to estimate levels of ambient air pollutants at unmeasured locations. Previous studies that have used kriging to map air pollution levels include: sulfur dioxide in Instanbul, Turkey [5], ozone in Atlanta [6,7] and northern Georgia [8], and particulates across the entire U.S. [9] and in Beijing, China [10]. Kriging has also been used in a range of epidemiologic studies of criteria pollutants to examine exposures to NO_2 _among pregnant women in Spain [11] as well as associations between: particulates and ozone and mortality in Los Angeles [12], particulates and mortality in Hamilton, Ontario, Canada [13], particulates and ectopy in the U.S. [9], CO, NO_2_, CO_2 _and preterm birth in Korea [14], ozone and pediatric asthma exacerbation in Atlanta [6], and particulates and low birth weight in Korea [15].

Although kriging appears to have become a useful tool in studies of criteria pollutants, in part because of the availability of monitoring data due to regulatory requirements, a limited number of studies have applied these methods to HAPs. Several studies have used kriging in the exposure assessment of ambient air levels of radon [16-18], but fewer investigations have applied this method to volatile organic compounds (VOCs). Following an extensive ambient air monitoring campaign that was conducted over a two-week period, Miller et al. [19] recently applied ordinary kriging to examine the spatial variability of total VOCs and BTEX (benzene, toluene, ethylbenzene and xylene, combined) in Detroit, Michigan (U.S.) and Windsor, Ontario (Canada). However, the usefulness of kriging to predict annual benzene levels using routine monitoring data, as might be needed in epidemiologic studies of health effects such as cancer, has not been fully explored. In contrast, data from the Assessment System for Population Exposure Nationwide (ASPEN) model, generated from the U.S. EPA has been applied in epidemiologic studies. Previously, for example, we conducted a study of childhood cancer in the Houston metropolitan area that used the ASPEN modeled estimates in the exposure assessment and found increased rates of childhood leukemia among census tracts with the highest levels of benzene [20]. Harris County, in which Houston is located, is home to a large number of petrochemical industries and is the fourth largest metropolitan area of the U.S., with a dense network of roadways. It is also one of the most closely monitored cities in the nation [21]. Given our previous epidemiologic finding, and in light of the fact that the ASPEN modeled estimates are only available for select years, we were interested in whether existing monitoring data could be used to provide additional information regarding exposure assessment of benzene. Hence, we conducted a study to apply kriging to predict ambient air levels of benzene for the years 1998 to 2000 at unmonitored locations in Harris County and to assess the degree of correspondence between the census tract-level kriged benzene levels and estimates of benzene obtained from the ASPEN model.

## Methods

### Data sources

The modeled data used in this analysis were from the 1999 U.S. EPA NATA project, which was undertaken to evaluate air toxics across the U.S [22]. As stated by the U.S. EPA, two uses of this assessment are to "provide a starting point for local-scale assessment" and to "inform monitoring programs" [23]. In 1999, NATA used the ASPEN model, a complex dispersion model, to estimate annual average concentrations of benzene and 176 other HAPs for each census tract in the contiguous U.S. and Puerto Rico. The 1999 ASPEN model incorporated meteorologic data, emissions data, and determinants of ambient air pollutant concentrations such as: rate, location and height of release, reactive decay, deposition, and secondary formation [24]. The 1999 ASPEN model also incorporated monitoring data to estimate the background concentrations (i.e., ambient air concentrations that result from emissions from natural sources, long-term transport from sources more than 50 km away, and emissions from unknown sources) [25].

**Figure 1:**
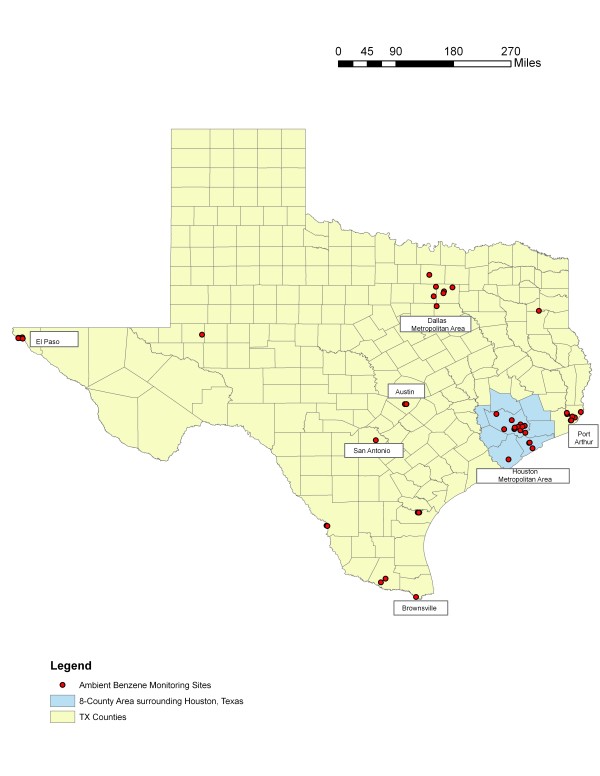
**Ambient Benzene Monitoring Locations in Texas**.

The monitoring data that were used in the present study were collected by the Texas Commission on Environmental Quality (TCEQ) and reported to the U.S. EPA Air Quality System (AQS) [26]. Although our interest was on the Houston metropolitan area in Harris County, we attempted to include monitoring data from surrounding counties to add to the exposure assessment. Of the seven counties surrounding Harris County, additional monitoring data were available only from Galveston and Brazoria counties. We restricted our analyses to data collected during the years 1998-2000 to facilitate comparisons to the 1999 ASPEN data. During this period, there were a total of 17 monitors that collected benzene measurements: 13 in Harris County, three in Galveston County, and one in Brazoria County. To increase stability of the estimates of the parameters of the semivariogram (see below), it became necessary to expand the number of monitoring sites to include benzene measurements collected at an additional 38 sites across Texas. Most of the additional monitors were located in urban areas including Dallas, San Antonio, Austin, El Paso, and Port Arthur (see Figure 1). The furthest monitor from the Houston metropolitan area was located in El Paso, approximately 750 miles west. These monitors collected 24-hr integrated samples with a sampling regimen of once every six days except for two sites that collected samples every 12 days and one site that collected samples daily for half the year and every six days for the remaining six months. These monitors used canister samples to collect benzene measurements and the method used to analyze the samples are detailed elsewhere [27]. For all 55 monitoring sites, on average, there was 77% complete data in 1998 and 1999, and 83% complete data in 2000. For the subset of 17 monitoring sites in the Houston metropolitan area, there were, on average, 80% complete data in 1998, 81% complete data in 1999, and 86% complete data in 2000 (data not shown).

### Data analysis

#### Kriging

Kriging is a statistical technique developed in geostatistics for optimal spatial prediction at unobserved locations [28]. Kriging requires that the parameters of a theoretical semivariogram function, which describe the spatial autocorrelation structure, be estimated from observed data and then uses information from observations at nearby locations along with the spatial structure to interpolate levels at unmonitored locations. The semivariogram is characterized by three parameters: the nugget effect, the sill, and the range. The nugget is the value of the semivariogram function at a distance of zero, the sill is the value at which the semivariogram levels off, and the range is the distance that corresponds to the sill. The difference between the sill and the nugget is often referred to as the partial sill.

We assessed both universal and ordinary kriging, which assume a non-stationary and stationary process, respectively, to determine the most appropriate model in our application. After computing annual averages of 24-hour concentrations for each monitoring site, we visually inspected a 3-dimensional plot of ambient benzene levels based on all sites in Texas, which provided no clear evidence of non-stationarity. We generated empirical semivariograms using (1) the estimated annual benzene levels for ordinary kriging or (2) the residuals from the regression analysis (with a mean structure specified as the longitude and latitude coordinates of the monitoring sites) for universal kriging. In both cases, a spherical model fit well to the empirical variograms. The equation for the spherical model of the semivariogram [28] is shown in Equation 1 below.(1)

In Equation 1, h is the distance between monitoring sites and θ = (c_0_, c_s_, α_s_)' where c_0 _represents the nugget effect(c_0 _≥ 0), c_s _represents the partial sill (sill minus the nugget effect)(c_s _≥ 0), and α_s _represents the range (α_s _≥ 0).

For both kriging models, we fit the theoretical semivariogram using nonlinear regression and, with the monitoring data from the 17 monitors in Harris, Brazoria, and Galveston Counties, used the model estimates to generate kriged values for each of the 649 census tracts in Harris County. Because three monitoring sites in Harris County were not in operation for all three years (one site was not operative in 2000, one site was not operative in 1998, and one site was not operative in 1998 or 1999), there were a total of 47 data points used for the kriging analysis. The parameter estimates of the trend obtained from the universal kriging model did not differ significantly from zero and similar results were obtained under both models (e.g., the mean, median, and mean standard error of the kriged benzene values were 0.745 ppbV, 0.741, and 0.354 ppbV for ordinary kriging and 0.757 ppbV, 0.759, and 0.383 ppbV for universal kriging, respectively). Moreover, we detected no difference in the residuals from the universal and ordinary kriging models using a Bland-Altman analysis [29] (results not shown). Because none of these results provided evidence of non-stationarity, we conducted all further analyses using the ordinary kriging model and report those results herein.

We performed two additional analyses. First, to determine how using the larger monitoring network for the variogram selection might affect the kriging results, we conducted a sensitivity analysis that relied upon the complete network of 55 monitors to predict benzene levels in Harris County and compared the kriged values from this analysis to those values using the original network of 17 monitors from Harris, Galveston, and Brazoria Counties. Secondly, to evaluate the impact of using a monitoring network that is spread across a relatively large geographic area, we stratified the results according to the distance of the census tract centroid from the individual monitoring sites. If the centroid of the census tracts was within 5 miles of a monitoring site, then the census tract was classified as "near"; if the census tract centroid was farther than 5 miles from a monitoring site, then the census tract was classified as "far". This stratification resulted in 281 "near" census tracts and 368 "far" census tracts. We implemented a z-test to formally compare the kappa statistics generated for these two groups.

#### Comparative analysis of kriged and modeled benzene levels

The non-parametric Kolmogorov-Smirnov two-sample test [30] was used to compare the empirical cumulative distribution functions for the two sets of benzene air levels (kriged predictions and ASPEN estimates). We also estimated the Spearman rank correlation coefficient between the kriged predictions and ASPEN estimates for the 649 census tracts in Harris County. We further categorized the data into quartiles (ASPEN estimates: ≤ 0.509 ppbV, 0.510 ppbV - 0.618 ppbV, 0.619 ppbV - 0.835 ppbV, ≥ 0.836 ppbV; kriged predictions: ≤ 0.678 ppbV, 0.679 ppbV - 0.740 ppbV, 0.741 ppbV - 0.814 ppbV, ≥ 0.815 ppbV) and used weighted Kappa statistics to compare the agreement between the discretized levels.

All analyses were conducted in SAS (version 9.1; SAS Institute Inc., Cary, NC).

## Results

Table 1 presents selected percentiles of the empirical cumulative distribution functions of the predicted benzene levels from the ordinary kriging model, as well as the ASPEN estimates for the 649 census tracts represented in our study. The distribution of the ASPEN estimates appears wider than the distribution of the kriged predictions although the median values for each of the distributions appear similar. Further, the distribution of average benzene levels from the 17 monitoring sites in Harris, Galveston, and Brazoria Counties more closely resembles the distribution of the ASPEN modeled data than the kriged data (data not shown). The results from the Kolmogorov-Smirnov test indicated that the ASPEN and kriged distributions were significantly different from one another (p < 0.0001).

**Table 1:** Distribution of ASPEN and kriged values of benzene levels in 649 Harris County census tracts.

Percentile	1999 ASPEN Modeled Estimate (ppbV)	1998-2000 Kriged Predicted Value (ppbV)
Maximum	2.83	0.89

99%	1.78	0.88

95%	1.35	0.87

90%	1.09	0.85

75%	0.84	0.81

50%	0.62	0.74

25%	0.51	0.68

10%	0.44	0.64

5%	0.40	0.62

1%	0.34	0.60

Minimum	0.26	0.58

**Figure 2:**
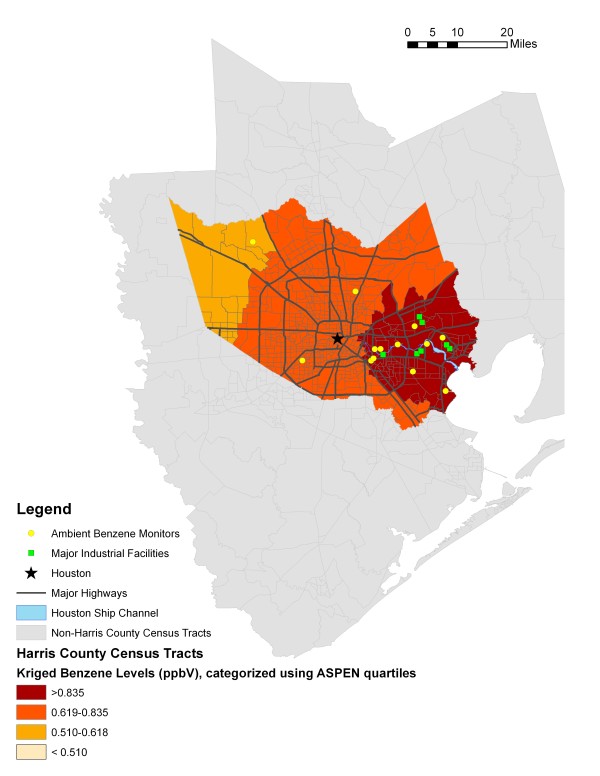
**Harris County Census Tract Level Ambient Benzene Levels based on Ordinary Kriging Model, using Monitoring Data from 1998-2000**.

**Figure 3:**
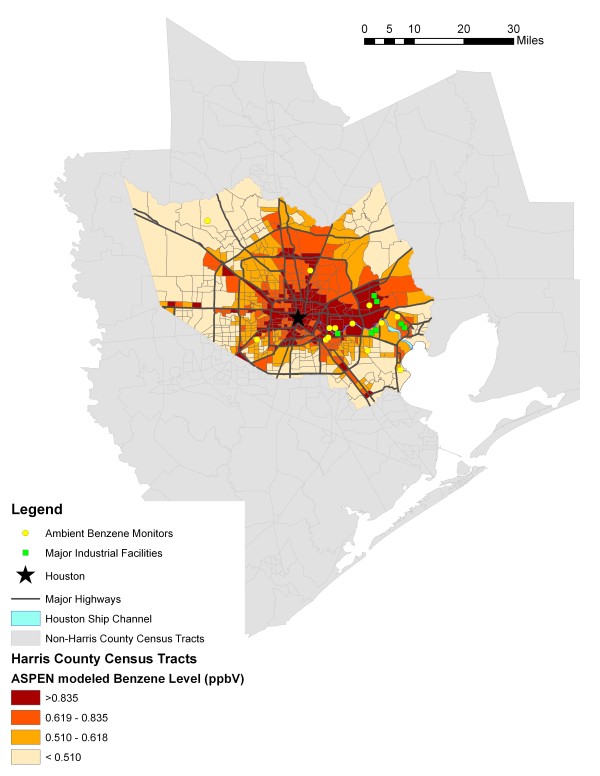
**Harris County Census Tract Level Ambient Benzene Levels based on the 1999 ASPEN Model**.

Figures 2 and 3 provide maps of Harris County and show the kriged values (generated using the 17 monitoring sites in Harris, Galveston, and Brazoria Counties) and ASPEN modeled estimates, categorized by quartiles. Both figures use the ASPEN quartile cut points to aid in the visual comparison of the two methods. Major highways and locations of industrial facilities that, together, contributed to 75% of the reported benzene emissions in 1999 are also represented. The facilities data were obtained from the EPA's Toxic Release Inventory [31]. The darkest areas represent census tracts with the highest estimated (or predicted) ambient levels of benzene, while the lightest areas represent census tracts with the lowest estimated (or predicted) levels. Note in Figure 2 that none of the census tracts' kriged benzene levels were categorized in the lowest ASPEN quartile.

The Spearman correlation coefficient between the ASPEN estimated and kriged values for benzene was 0.37 (p < 0.0001). Approximately 56% of the 162 census tracts classified in the lowest quartile of ambient air levels of benzene based on the ASPEN model were also classified in the lowest quartile based on the predictions from the kriging model (Table 2). Less than 30% of census tracts classified in the highest quartile by the ASPEN model were classified in the highest quartile for the kriging predictions. Similar results were obtained for the middle two quartiles. The weighted kappa coefficient was 0.26 (95% CI = 0.20, 0.31).

**Table 2:** Quartile classification of benzene levels in 649 Harris County census tracts, kriged values versus ASPEN estimates.

	1999 ASPEN Modeled Estimates			
	
1998-2000 Kriged Predicted Values	Low	Medium-Low	Medium-High	High
	No. (%)	No. (%)	No. (%)	No. (%)
Low	91 (56.2)	43 (26.5)	20 (12.4)	8 (4.9)

Medium-Low	11 (6.8)	66 (40.7)	50 (31.9)	35 (21.5)

Medium-High	23 (14.2)	14 (8.6)	51 (31.5)	74 (45.4)

High	37 (22.8)	39 (24.1)	41 (25.3)	46 (28.2)

**Total**	**162 (100.0)**	**162 (100.0)**	**162 (100.0)**	**163 (100.0)**

Note: kappa = 0.26 (95% confidence interval = 0.20, 0.31)

The sensitivity analysis comparing the kriged benzene values using the complete network of all 55 monitors in Texas versus the restricted network of 17 monitors in Harris, Galveston, and Brazoria Counties indicated similar results. The mean ambient benzene level using all 55 monitors was 0.73 (standard deviation (SD) = 0.10) while the mean ambient benzene level using the restricted network was 0.74 (SD = 0.08) and the mean benzene level from the ASPEN model was 0.71 (SD = 0.31). After stratifying by the "near" and "far" groups, the kappa statistic for the "far" census tracts was 0.26 (95% CI = 0.19, 0.33) while the kappa statistic for the "near" census tracts was 0.18 (95% CI = 0.09, 0.27). Although the magnitude of the kappa statistic for the "far" census tracts is greater than that for the "near" census tracts, they were not statistically significantly different (p = 0.3).

## Discussion

Our investigation made comparisons between a complex dispersion model developed by the U.S. EPA (ASPEN model) and a method of spatial interpolation (kriging) using routine monitoring data for benzene, a HAP. Overall, we found a lack of correspondence between kriged benzene predictions and ASPEN modeled estimates. The kriging model that we applied did not use data that could potentially affect variability in ambient benzene levels, including meteorology and source emissions from roadway traffic or industrial sites. The ASPEN model, in contrast, relies on more complex data sources, incorporating not only meteorological and pollutant source data, but also fate and transport data (including information about reactive decay of pollutants, deposition, and secondary formation) and monitoring data to estimate background concentrations of the pollutant of interest [25]. The discrepancies in the input data used by each method may contribute to the discordant results we observed. Although kriging has been used to interpolate ambient air levels of criteria air pollutants in exposure assessment and epidemiologic studies [6,9,11-15], few other studies have applied kriging to benzene [19,32].

The range of predicted benzene values observed in our study is smaller than that estimated from the ASPEN model. This may be due to the fact that kriging is a spatial smoothing tool that provides an optimal average over the region based on sampled observations. We also found poor agreement between the two metrics irrespective of whether the continuous or discretized values were evaluated. While significant, there was only modest correlation between the modeled and predicted values. Based on the quartile analysis, approximately 44% of census tracts classified by the ASPEN model as having the lowest ambient air levels of benzene were classified into a higher category using the predicted kriged results. Similarly, about 72% of census tracts in the highest quartile based on the ASPEN model were classified into lower categories using the kriging metric.

Measurements of ambient air pollutants using standard reference methods represent the optimal data source. Several studies have compared estimates from the ASPEN model to routine monitoring data and found reasonably good agreement between the two for ambient benzene levels [33-35]. Therefore, under the assumption that the ASPEN model yields reasonable estimates of ambient air pollutants at the census tract level, our results suggest that relying on kriged predictions instead would result in extensive misclassification had this exposure metric been used in an epidemiologic study. Although Houston, Texas is considered a closely monitored area [21], in our study, use of kriging that relies upon routine monitoring data for epidemiologic purposes or exposure assessment is still limited by a low ratio of monitoring sites to total land area. Given that we found similar agreement between the ASPEN modeled estimates and the kriged predictions for census tracts that were within 5 miles of a monitoring site as compared with census tracts further (>5 miles) from one or more monitoring sites, it appears that greater resolution in terms of the number of benzene monitors per area would be required for kriging, as has been suggested recently by the work of Cocheo and colleagues [36].

There has been success in applying kriging to predict ambient air levels of the criteria pollutants [9,11-15] which may be due, in part, to the regional nature of some of these pollutants as well as the relatively large monitoring networks utilized by many of these investigations. For example, the networks used in these studies ranged from 23 monitoring sites measuring PM_2.5 _and 42 ozone sites in the Los Angeles area [12] to 93 monitors measuring NO_2 _in Valencia, Spain to an average of 456 sites measuring PM_2.5 _across the U.S. [9]. Spatial variability over such expansive geographic regions are likely influenced more by meteorological and topographic factors than by specific emission sources and dispersion characteristics [37].

Another issue that may have affected the lack of agreement observed in the present study is the placement of monitors. Because the monitors are used primarily for regulatory purposes, they are not uniformly spatially located. The majority of the monitors used in our study are located near a large petrochemical complex in Houston (known as the Houston Ship Channel) and largely situated away from major transportation corridors in the county. As a result, the kriging predictions generally do not account for HAP levels arising from mobile sources, which make a significant contribution to ambient air levels of HAPs such as benzene [38,39]. In a recent study that applied kriging methodology to BTEX and other VOCs [19], after placement of 100 monitors across two cities in North America, the authors concluded that there was considerable intraurban variability in VOC levels in Detroit, Michigan (U.S.), where emissions of these chemicals are large and from similar sources to those in the greater Houston metropolitan area. Thus, we expect that interpolating ambient air levels of a localized (rather than regional) air pollutant will remain problematic in the absence of additional and more equitably distributed monitors across a geographic locale.

To obtain stable estimates of the theoretical semivariogram function, we used data from all ambient monitors in Texas. We assumed that the underlying spatial correlation between monitoring sites in our study area was similar to that of all monitoring sites in Texas. This is similar to others who have generated semivariograms for ozone and particulates [9,40] over five regions in the U.S., and for NO_2_, PM_10_, and O_3 _across the European Union [37]. Further, when we conducted a sensitivity analysis comparing the kriged predictions using the complete monitoring network of all Texas monitors versus only the 17 monitoring sites in Harris, Galveston, and Brazoria counties, the results were similar. At the time the present study was conducted, the ASPEN estimates were only available for 1999; thus, we restricted our use of the monitoring data to 1998-2000. Although we considered using only monitoring data from 1999, there were not enough data in this one year to accurately estimate the semivariogram. This is not likely to have introduced much error in our evaluation given that the annual mean benzene levels (mean ± 2SD) using all 55 monitoring sites in Texas for 1998 (N = 46), 1999 (N = 48), and 2000 (N = 46) were: 0.8 ppbV (0.1, 1.4), 0.9 ppbV (0.2, 1.5), and 0.8 (0, 1.6), respectively.

## Conclusion

This study compared predicted annual levels of benzene in ambient air to those generated by the ASPEN model to assess the value of kriging in epidemiologic investigations of HAPs. Due to the discrepancies found in these two methods, we feel that until improvements are made regarding the placement and number of monitors collecting HAP data, researchers interested in studying the health effects of these air pollutants must rely on data sources such as the ASPEN modeled estimates or other exposure models and innovative exposure assessment strategies such as generalized additive models or land use regression.

## List of Abbreviations

ASPEN: assessment system for population exposure nationwide; AQS: air quality system; BTEX: benzene, toluene, ethylene, and xylene; CO: carbon monoxide; CO_2_: carbon dioxide; GIS: geographic information systems; HAP: hazardous air pollutant; NAAQS: national ambient air quality standards; NATA: national air toxics assessment; NO_2_: nitrogen dioxide; O_3_: ozone; PM_10_: particulate matter ≤ 10 micrometers in diameter; VOC: volatile organic compound

## Competing interests

The authors declare that they have no competing interests.

## Authors' contributions

All authors have contributed to the overall direction of the research, and have read and approved the final version of the manuscript. KWW carried out the data analyses, participated in the interpretation of the data, and drafted and edited the manuscript. ES conceived of the study, participated in the interpretation of the data and drafted and edited the manuscript. DL provided statistical expertise related to kriging and interpretation of the data. ALC participated in interpretation of the data and edited the manuscript.
